# Household Air Pollution (HAP) and Cancer: What (HAP)pens Next?

**DOI:** 10.4172/2161-105X.1000189

**Published:** 2014-06-17

**Authors:** H. Dean Hosgood, Qing Lan, Thomas Rohan

**Affiliations:** 1 Department of Epidemiology and Population Health, Albert Einstein College of Medicine, Bronx, NY, USA; 2 Division of Cancer Epidemiology and Genetics, National Cancer Institute, Bethesda, MD, USA

## What is known about Household Air Pollution?

Humans around the world rely on a diversity of energy sources to provide heat for cooking and home comfort. Each of the energy sources utilized has a series of impacts on the environment and human health from extraction or harvest through combustion. At the household level, fuels that are considered to be clean, including gas, oil, and purchased electricity, yield lower levels of particulate emissions. Solid fuels including coal, wood, and other forms of biomass result in incomplete combustion which increases levels of known and suspected carcinogens, such as Polycyclic Aromatic Hydrocarbons (PAHs) and particulate matter (PM2.5) within homes. Household Air Pollution (HAP) consists of combustion by-products attributed to in-home solid fuel use.

The health impacts of HAP have been studied for several decades. The International Agency for Research on Cancer (IARC) classified HAP from coal as carcinogenic to humans (Group 1) [1]. In addition, indoor emissions from biomass, primarily wood, have been classified as probably carcinogenic to humans (Group 2A). Recently, the Global Burden of Disease (GBD) 2010 study brought together over 500 investigators throughout the world to determine the deaths and disability-adjusted life years attributed to 67 disease risk factors. Among these risk factors, HAP attributed to solid fuel use was deemed the 4^th^ leading risk factor for disease in the world, responsible for more than 3.5 million deaths per year [2].

In the aggregate, about half of the world's population, or 3 billion people, experience exposures to HAP [3]. HAP is a ubiquitous public health concern in developing countries where solid fuel use is common place. The level of concern in developed countries is also rising as people shift from cleaner fuel sources to cheaper, more local solid fuel options such as wood. When these factors are considered together, the global prevalence of exposure and the subsequent burden of disease are anticipated to increase over the coming decades.

The Xuanwei Cohort Study of 42,422 residents initially found that among (PAH-rich) smoky coal users, changing from an unvented stove to a stove with a chimney was associated with a significant reduction in the lung cancer incidence for both men and women [4]. Further analyses of the cohort also found that changing to a portable stove was associated with a significant reduction in lung cancer mortality for men and women [5]. These findings provide evidence that improving methods of solid fuel utilization in the home can have an impact on the global burden of disease.

A population-based case–control study in Xuanwei, consisting of 498 lung cancer cases and 498 controls, found marked heterogeneity in risk estimates for specific subtypes of smoky coal, suggesting that in Xuanwei and potentially elsewhere, the carcinogenic potential of coal combustion products can exhibit substantial local variation by specific coal source [6]. The risk of lung cancer associated with coal use extends beyond studies in Xuanwei. A meta-analysis of over 10,000 cases and 10,000 controls from mainland China, Taiwan, India, Africa, Europe, and the United States confirmed that significant increased risk was observed not only in Southwestern China, which includes Xuanwei, but in all other regions of mainland China where studies were conducted [7].

## What Remains to be Determined about Household Air Pollution?

In addition to the outstanding question of biomass's pulmonary carcinogenicity, other research questions have been posed by a recent panel of HAP experts convened by multiple United State's Federal Agencies [8]. One of this group's top research priority recommendations was to determine the associations between HAP and cancers other than the lung. Other important areas of research proposed include evaluating the genetic susceptibility to HAP-attributed cancers and determining if routes other than inhalation may play a role in HAP-attributed cancers.

While the research proposed by this expert panel into the risks associated with HAP and the underlying mechanism(s) is of importance, additional research must also focus on the mitigation and elimination of HAP exposures in the home in order to prevent cancer outcomes. For example, research is needed to determine the economic and cultural influences that help or hinder the effectiveness of and adherence to clean cooking stove initiatives at the personal, regional, and global levels.

## What (HAP)pens Next?

Research is critically needed to reduce the global disease burden from preventable HAP exposures. In the short term, research should leverage existing epidemiological studies to determine if cancer sites other than the lung are associated with HAP exposures, and if stove changes or interventions that minimize HAP exposures lead to a reduction in cancer risk and mortality. Efforts are underway in the Asian Cohort Consortium (ACC) [9], which was designed to elucidate the relationship between environmental exposures and the etiology of disease through a cohort of at least one million healthy people in Asia, to explore if HAP is associated with risk of cancer at other anatomic sites. In addition, follow-up analyses of the Xuanwei Cohort Study are exploring the impact that stove change has on cancer incidence and mortality. To the best of our knowledge, the Xuanwei Cohort is the only cohort to evaluate stove change and cancer outcomes. Finally, to help elucidate the underlying mechanism(s) of HAP-induced lung cancer, HAP-gene interactions are being analyzed in the Female Lung Cancer Consortium in Asia (FLCCA), which consists of over 6,600 cases and 7,400 controls from epidemiological studies of lung cancer restricted to never smoking female lung cancer cases and never smoking female controls [10].

These initiatives to answer the outstanding research questions relating to HAP are necessary for quantifying the impact of mitigating the human and environmental impacts experienced by communities relying on solid fuels; however, additional, new research initiatives that go beyond the existing studies are also urgently needed. For example, in the long term, prospective evaluations of the effectiveness of and adherence to fuel and stove improvements are necessary. This could be accomplished by either randomized intervention trials or pre- and post- exposure assessment and epidemiological studies in communities who undergo stove and/or fuel changes. Such evaluations must consider factors affecting the adaption of fuel and stove improvements, such as fuel cost and availability as well as stove cost and availability. While the methodological, ethical, cultural, and other considerations of such studies are beyond the scope of this commentary, it is clear that this research is critical for alleviating the tremendous burden of disease experienced by people as a result of simply trying to cook their meals and heat their homes.
